# Fitness, Fatness, and Academic Attainment in Male Schoolchildren from a Soccer Academy

**DOI:** 10.3390/ijerph19053106

**Published:** 2022-03-06

**Authors:** Souhail Hermassi, Thomas Bartels, Lawrence D. Hayes, René Schwesig

**Affiliations:** 1 Physical Education Department, College of Education, Qatar University, Doha 2713, Qatar; 2 Sports Clinic Halle, Center of Joint Surgery, 06108 Halle (Saale), Germany; thomas.bartels@sportklinik-halle.de; 3 School of Health and Life Sciences, University of the West of Scotland, Glasgow G72 0LH, UK; lawrence.hayes@uws.ac.uk; 4 Department of Orthopaedic and Trauma Surgery, Martin-Luther-University Halle-Wittenberg, Ernst-Grube-Str. 40, 06120 Halle (Saale), Germany; rene.schwesig@uk-halle.de

**Keywords:** soccer, anthropometrics, body fat, academic attainment, physical fitness, GPA

## Abstract

This investigation explored the association between anthropometric measures, fitness, and academic attainment (mathematics and science grade point average [GPA]) in male schoolchildren from a soccer academy. Thirty-one males (age: 10.3 ± 1.19 years; body mass: 41.7 ± 6.5 kg; height: 1.43 ± 0.07 m; body mass index (BMI): 20.2 ± 2.8 kg/m^2^) participated. Body mass, body fat percentage (%BF), and BMI were used as measures of anthropometry. The Yo-Yo Intermittent Recovery Test (level 1), squat and counter-movement jumps (SJ and CMJ), static balance, 10 and 15 m sprint, and a T-half test for change-of-direction (CoD) performance were used to measure fitness parameters. The GPA of mathematics and science determined academic attainment. All physical performance tests showed excellent relative reliability. ICC was between 0.87 (10 m sprint) and 1.00 (15 m sprint, CMJ). Regarding correlations between fatness and academic attainment, we found three correlations of practical value (r > 0.5), but only for mathematics (BMI: r = 0.540, subscapular skinfold: r = 0.589, body fat: r = 0.560). Mathematics was relevantly correlated with 15 m sprint (r = 0.574) and Yo-Yo IR1 test (r = 0.770). Only static balance (r = 0.428) did not reach the relevance criteria (r > 0.5). Science only showed large correlations with static balance (r = 0.620) and Yo-Yo IR1 test (r = 0.730). In conclusion, fatness and fitness are related to academic attainment in schoolchildren. In addition, except for static balance, all physical performance parameters were relevantly (r > 0.5) correlated with mathematics.

## 1. Introduction

Talent identification for soccer is of high importance for professional clubs, since there is a pervasive belief that this improves likelihood of an elite career for individuals [1,2]. Success in team sports is dependent upon several performance characteristics which are either sport-specific or transferable, i.e., technical, tactical, physiological, and psychological performance [3,4,5]. In childhood, players require development in several performance aspects in order to prosper and reach elite status [6]. In addition, there are convincing data that soccer performance and/or development requires information processing to perform in complex and rapidly changing contexts [6,7], emphasizing the importance of cognitive skills in soccer performance.

Therefore, soccer skill sets entail not only motor skills, but also cognitive control, comprised of core and higher-level cognitive functions such as inhibitory control and decision making [8,9]. Several past studies have separately explored either cognitive functions or motor skills in their separate associations with the success of top-level soccer players or, more generally, in characterizing high performance [10,11], comparing top and amateur level players among both adolescent and adult research participants.

During a 90 min match, elite youth soccer players complete intermittent running and commonly perform high-speed actions over short distances (i.e., sprinting, acceleration, and deceleration), and their particular movements are associated with soccer-specific actions such as tackling, defending, or creating space during possession, with sprints being the most common action before scoring a goal [12,13,14]. Other aspects of successful performance in team sports such as handball, rugby, volleyball, and basketball depend on the cooperation of team players to score more goals/points than the opposing team. All these team sports involve brief periods of high-intensity activity interspersed with lower-intensity activities that support play as well as provide brief recovery opportunities [15]. Concerning fatness, recent studies have suggested that anthropometric measurements might be related to physical fitness components in team sports [16]. For example, high body mass and %BF measurements are related to poor muscle power in soccer [17], basketball [18], and handball players [16].

There is a well-described positive association between academic attainment and physical fitness [19,20,21,22,23,24,25], which justifies school-based physical activity programs with the joint aim of increasing physical fitness and academic attainment in schoolchildren. Specifically, the positive relationship between cardiorespiratory fitness and academic achievement has attracted considerable attention in the last decade [21,22,23,24,26]. However, other fitness components such as agility, strength, and power and their relationship with academic achievement are less well researched [22,23], and therefore, associations between cognitive health and muscular fitness require further exploration [27].

Physical activity is associated with cognitive abilities (e.g., memory, executive function, attention, processing speed, and language processing) in adolescents [25,27,28,29]. Appropriate cognitive functioning permits adaptation environments and appropriate psychosocial development and mental health [27,28,30]. Cognitive performance is seemingly plastic, and recent meta-analytical evidence from 20 studies reports improved cognitive performance across several domains following body mass loss interventions [31].

However, sedentary behavior has become a growing public health concern, especially since it has been identified as a risk factor for health in youth. In fact, the amount of sedentary time in 0–12-year-old children ranges from 3.2 to 9.2 h a day [32]. Children spend up to half of their after-school period with sedentary behaviors (41–51%; 5–12 years), a number that increases with adolescence (57%; 12–18 years) [33]. In addition, research shows that elevated screen media use is a risk for health, as it has been associated with most of the previously investigated health aspects [34], especially with obesity [34,35]. Similar to overall sedentariness, there is a high prevalence of high screen time in children already [36]. It has been shown that children spend up to 2.7 h watching TV per day [32]. Most consistently, higher total sedentary behaviors, including leisure screen use, are negatively associated with all academic performance [37].

Several studies have intimated that children who are more physically active exhibit better academic attainment than those who are physically inactive [22,23,38]. However, associations with academic attainment and measures of fitness like strength or speed are less researched, and there are calls for additional investigation in this field [39]. A difficulty in this research area is conflating physical activity and physical fitness. This begs the question as to the importance of volume of physical activity, or the physiological adaptation resultant from the physical activity (i.e., enhanced fitness). One method of mitigating this ambiguity and conflation is to examine participants from one sport of the same performance level, or even more preferentially, the same sports team, as between-subject physical activity variation would be ameliorated due to ubiquitous training volumes and intensities as they partake in the same training and match play.

Aerobic fitness is presumably the strongest correlate of academic attainment, and students who maintain fitness throughout high school outperform those who lose fitness [40]. Importantly, the magnitude of effect for this phenomenon is sufficient to make a difference to the quality of university to which children are accepted.

To date, the majority of results from research support previously published results concerning the negative impact of high %BF on both aerobic and anaerobic fitness related components in 10- to 12-year-old school-aged team handball players in Qatar [41]. Previous reports confirm that correlations exist between fatness, physical fitness, and academic performance in pre-pubertal handball children in Qatar [22,23,24]. In addition, the results of Hermassi et al. [22] show that both BMI and %BF were negatively correlated with academic performance of schoolchildren handball players. More research is required to test associations of different components of physical fitness (i.e., muscular strength, aerobic fitness, speed, agility, and postural stability) with academic performance [42] in specific populations (i.e., soccer players). Using one population for this research question controls for physical activity levels to some extent, as all players within one team in a sport will likely complete the same amount of training.

To date, few studies have been conducted in youth soccer concerning physical fitness, fatness, and academic attainment in male schoolchildren. With talent identification and development in mind, it would seem relevant to investigate various cognitive processes in relation to fatness and physical performance. Therefore, the aim of the present investigation was to test for associations between fatness, different components of physical fitness, and academic attainment in male schoolchildren from a soccer academy (in an attempt to control for physical activity levels) in Qatar. We hypothesized a priori that relationships would be evident between measures of physical fitness, fatness, and academic attainment.

## 2. Materials and Methods

### 2.1. Participants

Thirty-one youth-academy-level schoolchildren from a soccer academy in Doha, Qatar participated (age: 10.3 ± 1.2 years; body mass: 41.7 ± 6.5 kg; height: 1.43 ± 0.07 m; body fat: 18.5 ± 3.8%). Participants had ≥2 years soccer playing experience and self-reported no musculoskeletal injuries in the 4 weeks preceding the study. Participants undertook soccer training 2–3 times per week (~3.8 h/week), which consisted of motor skills, technique, and tactical training. 60% of session time focused on motor skills while 40% of session time focused on basic team soccer techniques. Participants also undertook compulsory weekly school physical education lessons (~45 min of mostly ball games). Participants abstained from exercise for the 24 h preceding testing, and informed consent or assent was provided prior to enrollment. This investigation was conducted in line with the Declaration of Helsinki. Moreover, this cross-sectional study was approved by institutional review board of Qatar University (QU-IRB 1610-FBA/21).

### 2.2. Procedures and Evaluations

Testing was completed on an outdoor soccer field, from 17:00–19:00 p.m., to minimize the effect of diurnal variation, ≥3 days after a competitive match. Participants maintained habitual nutrition, and they had also abstained from caffeine-containing beverages on the testing day. Participants were ≥4 h postprandial when attending testing sessions. Tests were 10 and 15 m sprints, countermovement jump (CMJ), squat jump (SJ), medicine ball overhead throw, standing stork test, and the Yo-Yo intermittent recovery test-1 (Yo-Yo IR1).

A general warm-up consisting of 5 min low-intensity running, 3 × 15 m progressive accelerations, a maximal 20 m sprint, and dynamic stretches and throws as previously described [43] preceded all tests. Testing was conducted over five days, in the same order for all participants.

**Figure 1:**
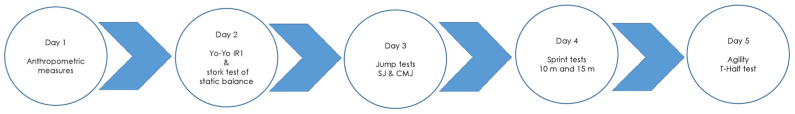
Timeline of testing procedures and evaluations.

Determination of BMI and percentage body fat was conducted on day one. On day two, the Yo-Yo IR1 and stork test of static balance were completed. On day three, the CMJ and SJ were completed. On the fourth day, sprint tests (10 and 15 m) and handgrip force were assessed, and finally, on the fifth day, the agility T-half test was assessed (Figure 1). Tests (except anthropometry and Yo-Yo IR1) were repeated two weeks later to allow determination of test–retest reliability, with the second set of values used for statistical analysis.

### 2.3. Anthropometry

Stature and body mass were measured using a portable digital scale accurate to 0.1 cm and 0.1 kg, respectively (Tanita Body Fat Analyzer; model TBF 105; Tanita Corporation of America, Inc., Arlington Heights, IL, USA). Subsequently, BMI was determined by dividing body mass by stature squared (kg·m^2^). Skinfold body fat assessment was completed in duplicate with Harpenden calipers to the nearest 0.1 mm (Baty International, Burgess Hill, Sussex, UK). If duplicate readings exceeded 2 mm difference, a third reading was recorded, with the mean of the closest two readings taken for statistical analysis. Body fat percentage was estimated using the four-site method and sex- and age-specific equations [44], which has previously been reported in young athletes [22,23]. The sites examined were biceps, triceps, subscapular, and suprailiac, using the equation below:

% Body fat = (4.95/(Density-4.5)) × 100

Where Density = 1.162–0.063 (LOG sum of 4 skinfolds).

### 2.4. Maturity

Somatic maturity was estimated using the distance from peak height velocity (Y-PHV) via sex-specific equations which incorporated measures of body mass, stature, leg length, and sitting height [45].

### 2.5. The Yo-Yo Intermittent Recovery Test Level 1

The Yo-Yo IR1 was conducted in accordance with the description of Krustrup et al. [46]. Twenty-meter shuttle runs of increasing velocity were performed until fatigue, with 10 s of active recovery (2 × 5 m of jogging) between each run. The test ended when objective criteria (the participant twice failing to reach the front line in time) or subjective criteria (the participant feeling unable to continue at the required velocity) were met. Total distance run during the test was taken for statistical analysis.

### 2.6. Sprint Tests

Ten- and fifteen-meter sprints were conducted as previously described [22] using paired photocells (Racetime 2 SF, Microgate, Italy). Three trials interspersed by 6–8 min of rest were performed, and the best value (i.e., least number of seconds) was used for statistical analysis.

### 2.7. Vertical Jumps

CMJ and SJ were performed as previously described [22] using the OptoJump photoelectronic system (Optojump Next, Microgate, Italy). Four trials interspersed by 30 s of rest were performed, and the best value (i.e., greatest jump height) was used for statistical analysis.

### 2.8. Static Balance Performance

The stork balance test was conducted as previously described [47]. The outcome variable used for statistical analysis from this test was the number of seconds balanced.

### 2.9. Change of Direction (T-Half Test)

Electronic timing sensors (photocells, Kit Racetime 2 SF, Microgate, Italy) were employed to record T-Half tests as previously described [22]. Participants performed two trials with a 3 min break between trials, and the best trial was used for statistical analyses [48].

### 2.10. Academic Attainment

Academic attainment was evaluated through school records. Academic attainment consisted of actual GPA and score (0 to 100) as endorsed in the Qatar State in mathematics and science from the second semester of the academic year 2020–2021. Mathematics and science were the only subjects included in the analyses, because fitness is beneficial for subjects more reliant on executive cognition, such as the subjects mentioned above, and this in turn determines academic achievement [23,49].

### 2.11. Statistical Analysis

Statistical analyses were performed on SPSS version 28.0 for Windows (SPSS Inc., IBM, Armonk, NY, USA). Prior to analysis, data were tested for normality (Shapiro-Wilk Test) and homogeneity of variance (Levene’s test).

Reliability was interpreted using intraclass correlation coefficients [50] and coefficient of variation (CV) over pairs of intra-participant trials [51].

Pearson’s product moment correlations were calculated and used to determine relationships between anthropometric and performance (physical and academic) parameters. A correlation (r) of <0.1, 0.1–0.3, 0.3–0.5, 0.5–0.7, 0.7–0.9, and >0.9, was considered trivial, small, moderate, large, very large, and almost perfect, respectively [52]. r^2^ > 0.5 (explained variance > 50%) was defined as relevant and marked in bold.

Regarding the sample size of *n* = 31, the critical value for the product-moment-correlation based on a two-sided t-test and a = 5% is r = 0.345 [53].

Data are reported as mean ± standard deviation (SD).

## 3. Results

### 3.1. Normal Distribution and Homogeneity of Variance

Seven variables (leg length: *p* = 0.022; suprailiac: *p* < 0.001; sprint 10 m: *p* = 0.023; CMJ: *p* = 0.031; static balance: *p* = 0.007; mathematics: *p* = 0.014; science: *p* = 0.005) were not normally distributed.

### 3.2. Intrarater Reliability

All six performance tests showed excellent relative reliability (ICC ≥ 0.75). ICC moved between 0.87 (sprint 10 m) and 1.00 (sprint 15 m, CMJ). Apart from the balance test (CV = 13.7%), all variables had excellent absolute reliability, with CV < 5% (Table 1).

Table 2 and Table 3 provide all descriptive data regarding all parameters.

### 3.3. Correlation between Fatness and Academic Attainment

**Figure 2:**
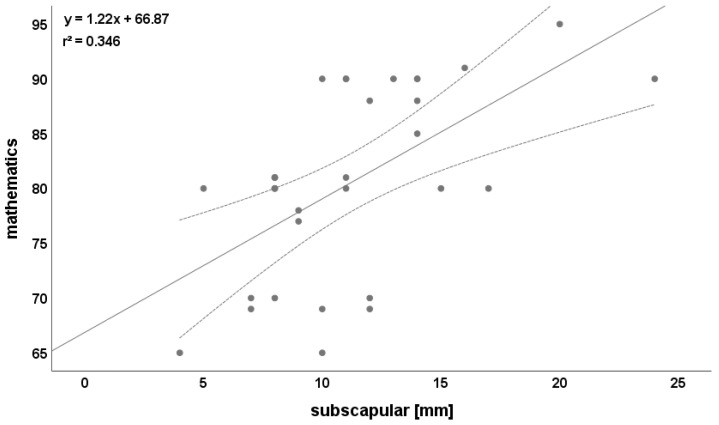
Relationship between subscapular skinfolds and mathematics. Please note that one dot can represent multiple participants.

Relevant correlations (r > 0.5) for mathematics were found with BMI (r = 0.540), subscapular skinfold (r = 0.589, Figure 2), and body fat (r = 0.560). Science was not correlated on a relevant level with any fatness parameter (Table 4).

**Figure 3:**
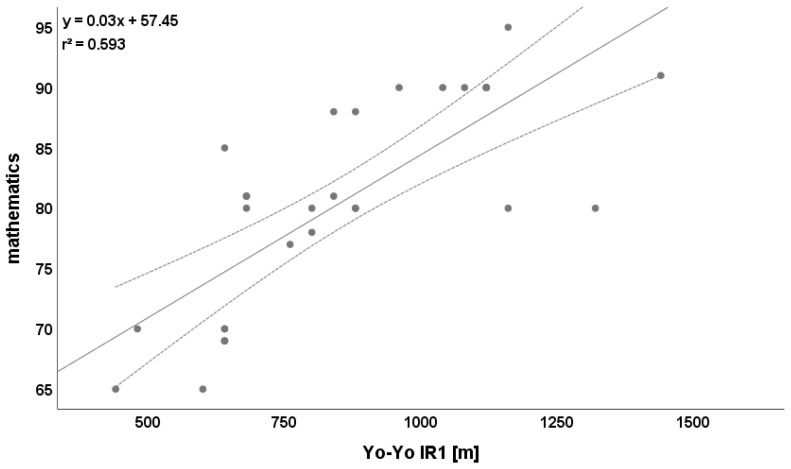
Relationship between Yo-Yo IR1 test and mathematics. Please note that one dot can represent several subjects.

Except for static balance, all physical performance parameters were relevantly correlated with mathematics (r = 0.428). The amount of correlation ranged from r = 0.574 (sprint 15 m) to r = 0.770 (Yo-Yo IR1 test, Figure 3, Table 4).

**Figure 4:**
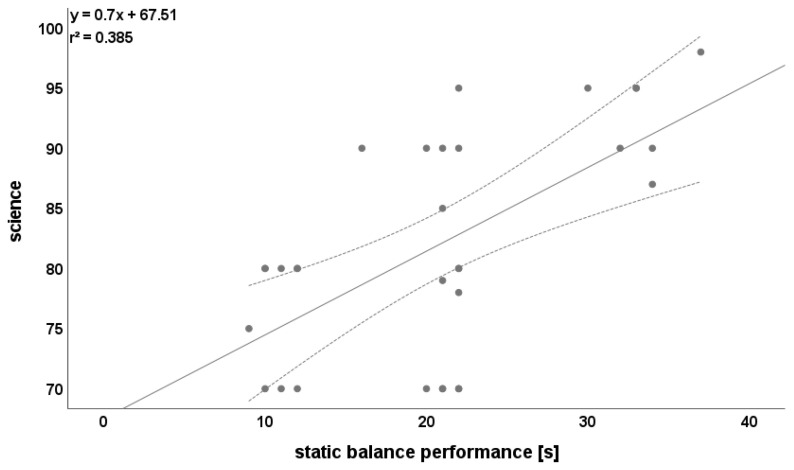
Relationship between static balance and science. Please note that one dot can represent multiple participants.

**Figure 5:**
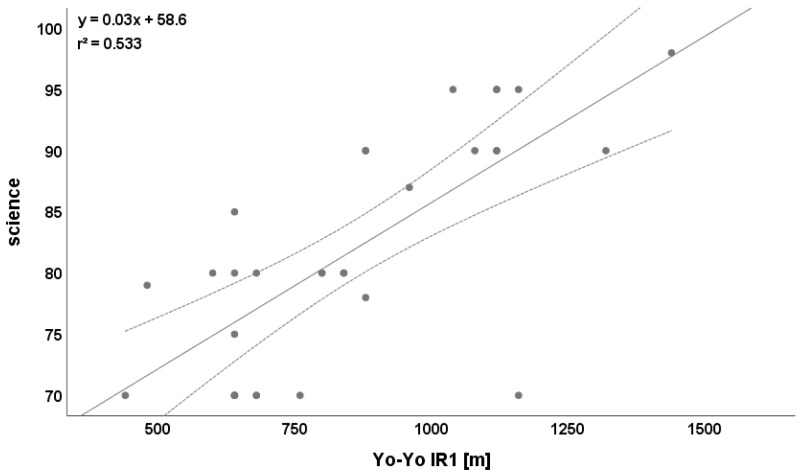
Relationship between Yo-Yo IR1 test and science. Please note that one dot can represent multiple participants.

Science was highly correlated with static balance (r = 0.620, Figure 4) and Yo-Yo IR1 test (r = 0.730, Figure 5).

## 4. Discussion

This investigation resulted in correlations between fatness and academic attainment in male soccer-playing schoolchildren. BMI and %BF were both inversely related with mathematics attainment (but not other subjects). Moreover, mathematics performance was more strongly correlated with %BF than BMI, suggesting adiposity rather than excess body mass per se is negatively associated with academic attainment. Science was not correlated with any fatness estimate (%BF or BMI). In terms of direct fitness measures and academic attainment, the Yo-Yo IR1, jump performance, and CoD ability had a positive, linear relationship with academic attainment. These data suggest fitness is more closely related to academic attainment than anthropometry. Thus, maintaining normal body mass appears less important for academic attainment than fitness.

### 4.1. Relationship between Fatness and Academic Attainment

According to World Health Organization growth charts [54], children in the present study had normal BMI [55]. The negative associations between BMI and %BF with mathematics GPA in the present study supports the inference that children with greater adiposity exhibit poorer academic attainment [22,23,25,56,57]. Conversely, an association between BMI and academic attainment is not ubiquitous in previous investigations [58,59,60] as some studies reported no relationship [61,62]. A reason for this discrepancy could be similarities in academic attainment between highest and lowest quartiles of BMI, which would result in no linear association between these variables after covariate adjustment [22]. It is possible that an inverted-U pattern may exist whereby children of normal body mass exhibit the greatest academic achievement and those at either end of the BMI spectrum exhibit the poorest. However, the present findings are in line from the results of Castelli et al. [63], which demonstrated lower BMI associated with greater academic attainment. As %BF associated more strongly with mathematics than BMI, we suggest direct measurement of adiposity is superior to indirect estimates such as BMI, and this should be reflected in future investigations.

Mechanistically, our findings seem to underline the contention that poorer working memory is evident in students with greater adiposity because working memory is associated with academic attainment [25,64], mathematics and reading test scores [65,66], arts and science test scores [65], lower efficiency in conflict resolution, and poorer inhibitory control [67].

### 4.2. Associations between Physical Fitness and GPA

The results of the present investigation indicated that mathematics was correlated with physical fitness except static balance. Science only correlated with static balance and the Yo-Yo IR1 test. Thus, our data support previous cross-sectional findings which demonstrated fitter students had superior academic attainment [22,23]. Reasons for this are likely multi-factorial, but one fact could be that physical fitness is typically associated with improved overall health, which in turn associates with academic attainment [68]. Moreover, greater physical fitness improves attention and classroom behavior [21]. Finally, superior fitness positively influences mental health which would sequentially influence academic achievement.

Regarding aerobic performance and its association with GPA, the present study adds to the existing literature base which has also observed positive associations [22,68,69]. For instance, van Dusen et al. [70] reported aerobic fitness was the strongest fitness predictor of academic attainment which confirms previous investigations [61,69,70]. Our group has previously reported the Yo-Yo IR1 was positively correlated with science and mathematics in ~9-year-old handball players [22] which supports multiple previous studies [61,71], adding confidence the association is existent at several ages. In terms of mechanisms, exercise improves or maintains aerobic fitness which affects brain plasticity and cerebral blood flow [72] and is associated with superior cognitive abilities including executive function [73]. Moreover, physical fitness is positively related to perceived exercise and sport competence, which could be a possible indicator of intrinsic motivation [74]. The data presented herein emphasize the importance of aerobic fitness for cognitive abilities of schoolchildren.

Positive correlations between lower-limb muscular power (CMJ, SJ, 10 m and 15 m sprint and CoD ability) and mathematics were observed. Scarce research has been performed examining the associations between muscular strength and academic attainment, and with inconsistent results. However, Hermassi et al. [22] reported that CMJ and SJ were related to academic attainment in young handball players supporting previous data [75,76]. For example, Cadenas-Sanchez et al. [69] reported a correlation between lower-limb muscular strength (i.e., 1-RM leg press) and mathematics performance (Woodcock–Muñoz test battery). This positive association is consonant with previous cross-sectional studies in children [70]. Paradoxically, other studies have reported no relationship between muscular strength and academic attainment [39,62,71]. Conflicting results may be related to study sample characteristics, or different strength testing means (e.g., isokinetic dynamometry, free weights, body-mass-related tests), and different academic evaluations. Regardless, ambiguity remains and therefore further research is required to elucidate why divergent results are evident.

It has been demonstrated that high coordination skills during changes of direction (speed–agility), sprinting phases, left- and right-side shuffling, back pedaling run, and specific soccer strikes [77] were accompanied by an integration of cognitive functions (in this case, action planning) that allowed successful motor performance [9]. The conclusions of Lovecchio et al. [9] underline that both components (cognitive and motor skills) elicit successful performance in soccer among players in mid-childhood and not only among those adolescence [78]. However, performance identification might harness routines based not only on technical–tactical skills, but also on these aspects of cognitive functioning. Relying on open-skills, a successful soccer behavior requires sport-specific perceptual abilities (“game intelligence”) that correspond to the “cognitive functions” [9].

In the present investigation, no correlation existed between static balance and mathematics, but a correlation between science and static balance was observed. Mathematics correlated with 10 m sprint, 15 m sprint, SJ, CMJ, and CoD ability. The association between academic attainment and CoD ability corroborates the observation of van Dusen et al. [70] although explanatory mechanisms to explain this phenomenon remain unelucidated, and further investigation is required to elaborate on the association between agility and academic attainment.

### 4.3. Limitations

Body composition components (i.e., muscle, bone) exhibit individual temporal changes, which it is difficult to capture in a cross-sectional design. Participants were recruited from within a narrow age range, in an attempt to control for this. The lack of sociodemographic and socioeconomic variables is another limitation to acknowledge. Moreover, although the sample size was modest, characteristic of the sample (i.e., academy soccer players) provide a novel view of the associations studied, controlling for physical activity levels to some extent. Regardless, future investigations should incorporate sexual maturation assessment to increase robustness of maturation determination when examining relationships between anthropometry and physical performance in adolescents. Secondly, as the analyses conducted herein were observational correlations, it is difficult to attribute causality. However, implementing an intervention to test causation would require considerably greater resource commitment.

## 5. Conclusions

The present findings support the assumption that a correlations exist between fatness, fitness, and academic performance in schoolchildren. In conclusion, physical fitness (more than fatness) was related to academic attainment in schoolchildren who played soccer. Except for static balance, all physical performance parameters were correlated with mathematics. Based on associations presented here, it seems pragmatic to promote physical fitness in this age group. This would likely be done via exercise or physical activity, which in turn could increase physical fitness, and improve academic performance. Future studies with additional variables (e.g., lean mass and hormonal maturation status) are required to confirm these preliminary observations.

## Figures and Tables

**Table 1:** Fitness test data from two sessions (*n* = 31). Descriptive statistics and intrarater reliability are presented for each test. Intraclass correlation coefficient (ICC) ≥ 0.75 and coefficient of variation (CV) ≤ 10% marked in bold. Data are reported as mean ± standard deviation (SD).

Test	Session One (Mean ± SD)	Session Two (Mean ± SD)	ICC (95% CI)	CV (%) (95% CI)
10 m sprint (s)	2.29 ± 0.23	2.28 ± 0.21	**0.97** (0.94–0.99)	**2.2** (1.7–3.4)
15 m sprint (s)	3.39 ± 0.34	3.41 ± 0.34	**1.00** (0.99–1.00)	**0.9** (0.7–1.5)
Agility T-half test (s)	7.50 ± 0.92	7.83 ± 1.02	**0.93** (0.68–0.98)	**4.2** (3.2–6.5)
SJ (cm)	25.2 ± 6.5	25.7 ± 6.52	**0.98** (0.96–0.99)	**1.6** (1.2–2.5)
CMJ (cm)	28.2 ± 6.09	28.4 ± 5.99	**1.00** (0.99–1.00)	**1.5** (1.1–2.3)
Static balance (s)	20.8 ± 8.30	20.7 ± 7.60	**0.98** (0.97–0.99)	13.7 (10.7–23.1)

**Table 2:** Descriptive values (mean ± standard deviation (SD), range (minimum–maximum)) of all physical and academic parameters (*n* = 31).

	Mean ± SD	Range
**Physical Performance Parameters**		
10 m sprint (s)	2.29 ± 0.23	1.99–2.90
15 m sprint (s)	3.39 ± 0.34	2.50–3.99
Agility T-half test (s)	7.50 ± 0.92	5.81–9.00
SJ (cm)	25.2 ± 6.53	14.8–36.0
CMJ (cm)	28.3 ± 6.01	16.7–36.0
Postural balance (s)	20.8 ± 8.3	9.0–37.0
Yo-Yo IR1 (m)	862 ± 251	440–1440
**Academic Attainment Parameters**		
Mathematics	81 ± 9	65–95
Science	82 ± 9	70–98

**Table 3:** Descriptive values (mean ± standard deviation, range) of all anthropometric parameters (*n* = 31).

	Mean ± SD	Range
Height (m)	1.43 ± 0.07	1.27–1.60
Body mass (kg)	41.7 ± 6.45	25.0–55.0
Leg length (cm)	73.1 ± 3.81	68.0–80.0
Sitting height (cm)	75.2 ± 3.73	68.0–83.0
BMI (kg/m^2^)	20.3 ± 2.82	15.2–26.2
bicipital (mm)	10.3 ± 3.96	4.00–21.0
tricipital (mm)	13.8 ± 5.20	6.00–27.0
suprailiac (mm)	11.5 ± 5.58	5.00–30.0
subscapular (mm)	11.4 ± 4.24	4.00–24.0
Body fat (%)	18.5 ± 3.84	9.45–28.0
Y-PHV	2.67 ± 0.80	1.12–4.05

**Table 4:** Intercorrelation matrix for measure of anthropometrics, academic and physical performances. Relevant correlations (r < 0.5) marked in bold.

Intercorrelations (r) between Science/Mathematics and Anthropometrics																	
	Height	Weight		leg length	Sitting height		BMI	Bicipital		Tricipital		Suprailiac	Subscapular		Fat		Y-PHY
Mathematics	−0.041	0.436		−0.056	−0.243		**0.540**	0.427		0.314		0.476	**0.589**		**0.560**		−0.047
Science	−0.152	0.103		−0.124	−0.205		0.238	0.018		0.202		0.012	0.321		0.173		0.121
**Intercorrelations (r) between Science/Mathematics and Physical Performances**																	
	10 m sprint		15 m sprint			Agility T-half test			SJ		CMJ			Static balance		Yo-Yo IR1	
Mathematics	**0.604**		**0.574**			**0.722**			**0.624**		**0.577**			0.428		**0.770**	
Science	0.280		0.466			0.370			0.258		0.155			**0.620**		**0.730**	

## Data Availability

The raw data supporting the conclusions of this article will be made available by the authors without undue reservation.
